# Supplementary material for: Modeling glioblastoma heterogeneity as a dynamic network of cell states
Source: Mol Syst Biol. 2021 Sep 16;17(9):e10105. doi: 10.15252/msb.202010105 (PMC8444284; doi:10.15252/msb.202010105)
Supplement: Supplementary file 6 — Source Data for Figure 5 [file MSB-17-e10105-s004.zip › Figure5A_sourcedata/GSEA_3071/nosigdb_stateII.GseaPreranked.1622795151171/RICHARDS_NOWAKOWSKI_SCIENCE_2017_NIN1_UPREG.html]

Details for gene set RICHARDS\_NOWAKOWSKI\_SCIENCE\_2017\_NIN1\_UPREG[GSEA]

|  || Dataset | state23071 |
| Phenotype | NoPhenotypeAvailable |
| Upregulated in class | na\_neg |
| GeneSet | RICHARDS\_NOWAKOWSKI\_SCIENCE\_2017\_NIN1\_UPREG |
| Enrichment Score (ES) | -0.6150484 |
| Normalized Enrichment Score (NES) | -3.3634048 |
| Nominal p-value | 0.0 |
| FDR q-value | 0.0 |
| FWER p-Value | 0.0 |
Table: GSEA Results Summary

  

Fig 1: Enrichment plot: RICHARDS\_NOWAKOWSKI\_SCIENCE\_2017\_NIN1\_UPREG      
 Profile of the Running ES Score & Positions of GeneSet Members on the Rank Ordered List

  

| PROBE | GENE SYMBOL | GENE\_TITLE | RANK IN GENE LIST | RANK METRIC SCORE | RUNNING ES | CORE ENRICHMENT || 1 | CCNE2 |  |  | 76 | 1.121 | -0.0019 | No |
| 2 | ALYREF |  |  | 315 | 0.741 | -0.0501 | No |
| 3 | ACTG1 |  |  | 755 | 0.514 | -0.1519 | No |
| 4 | GART |  |  | 820 | 0.491 | -0.1604 | No |
| 5 | RND3 |  |  | 860 | 0.482 | -0.1628 | No |
| 6 | SACS |  |  | 1195 | 0.397 | -0.2401 | No |
| 7 | EIF1AX |  |  | 1198 | 0.397 | -0.2346 | No |
| 8 | NUP88 |  |  | 1302 | 0.378 | -0.2546 | No |
| 9 | PARP1 |  |  | 1431 | 0.351 | -0.2812 | No |
| 10 | DIMT1 |  |  | 1591 | 0.326 | -0.3159 | No |
| 11 | EFNA5 |  |  | 1624 | 0.320 | -0.3191 | No |
| 12 | LINC00342 |  |  | 1685 | 0.312 | -0.3293 | No |
| 13 | PSMG2 |  |  | 1723 | 0.307 | -0.3339 | No |
| 14 | EHBP1 |  |  | 2018 | 0.271 | -0.4032 | No |
| 15 | TSPYL4 |  |  | 2235 | -0.252 | -0.4533 | No |
| 16 | PPHLN1 |  |  | 2299 | -0.259 | -0.4651 | No |
| 17 | NBEA |  |  | 2334 | -0.263 | -0.4695 | No |
| 18 | RUNX1T1 |  |  | 2458 | -0.278 | -0.4960 | No |
| 19 | MTRNR2L1 |  |  | 2471 | -0.280 | -0.4948 | No |
| 20 | MAP9 |  |  | 2532 | -0.291 | -0.5053 | No |
| 21 | SRGAP2B |  |  | 2638 | -0.307 | -0.5269 | No |
| 22 | SMARCD1 |  |  | 2665 | -0.311 | -0.5286 | No |
| 23 | RNMT |  |  | 2805 | -0.336 | -0.5582 | No |
| 24 | ZFAND5 |  |  | 2855 | -0.345 | -0.5652 | No |
| 25 | MFN1 |  |  | 2859 | -0.346 | -0.5607 | No |
| 26 | GNPTAB |  |  | 2862 | -0.346 | -0.5559 | No |
| 27 | PCDH17 |  |  | 2924 | -0.360 | -0.5657 | No |
| 28 | ATP6V1G1 |  |  | 2968 | -0.367 | -0.5709 | No |
| 29 | MIAT |  |  | 2996 | -0.371 | -0.5720 | No |
| 30 | HIPK2 |  |  | 3029 | -0.379 | -0.5742 | No |
| 31 | PHF20L1 |  |  | 3143 | -0.412 | -0.5961 | No |
| 32 | CHMP1B |  |  | 3207 | -0.430 | -0.6053 | No |
| 33 | FXR1 |  |  | 3247 | -0.441 | -0.6083 | Yes |
| 34 | AP2M1 |  |  | 3272 | -0.448 | -0.6075 | Yes |
| 35 | SENP7 |  |  | 3274 | -0.448 | -0.6010 | Yes |
| 36 | PDZRN3 |  |  | 3276 | -0.449 | -0.5944 | Yes |
| 37 | PDZRN4 |  |  | 3279 | -0.450 | -0.5881 | Yes |
| 38 | HDAC9 |  |  | 3288 | -0.453 | -0.5832 | Yes |
| 39 | BEST3 |  |  | 3302 | -0.458 | -0.5794 | Yes |
| 40 | HIGD1A |  |  | 3310 | -0.460 | -0.5742 | Yes |
| 41 | ARID4B |  |  | 3330 | -0.467 | -0.5718 | Yes |
| 42 | ELAVL3 |  |  | 3341 | -0.472 | -0.5672 | Yes |
| 43 | MARCKS |  |  | 3369 | -0.487 | -0.5665 | Yes |
| 44 | B4GALT5 |  |  | 3372 | -0.487 | -0.5596 | Yes |
| 45 | ST8SIA4 |  |  | 3406 | -0.500 | -0.5602 | Yes |
| 46 | COL4A3BP |  |  | 3414 | -0.505 | -0.5543 | Yes |
| 47 | ARMCX3 |  |  | 3418 | -0.508 | -0.5473 | Yes |
| 48 | SYF2 |  |  | 3419 | -0.509 | -0.5396 | Yes |
| 49 | ARX |  |  | 3443 | -0.516 | -0.5375 | Yes |
| 50 | GLCCI1 |  |  | 3444 | -0.517 | -0.5296 | Yes |
| 51 | ARL4D |  |  | 3453 | -0.521 | -0.5237 | Yes |
| 52 | CDK6 |  |  | 3466 | -0.526 | -0.5187 | Yes |
| 53 | QKI |  |  | 3467 | -0.526 | -0.5107 | Yes |
| 54 | KLHL13 |  |  | 3486 | -0.534 | -0.5070 | Yes |
| 55 | STAT2 |  |  | 3496 | -0.539 | -0.5011 | Yes |
| 56 | FNBP1L |  |  | 3615 | -0.614 | -0.5212 | Yes |
| 57 | ANTXR1 |  |  | 3634 | -0.630 | -0.5161 | Yes |
| 58 | MEIS1 |  |  | 3651 | -0.648 | -0.5103 | Yes |
| 59 | BCL11B |  |  | 3656 | -0.651 | -0.5014 | Yes |
| 60 | GOLM1 |  |  | 3699 | -0.684 | -0.5014 | Yes |
| 61 | GAD1 |  |  | 3704 | -0.690 | -0.4919 | Yes |
| 62 | DLX6 |  |  | 3735 | -0.711 | -0.4886 | Yes |
| 63 | NRXN3 |  |  | 3738 | -0.714 | -0.4783 | Yes |
| 64 | ZNF704 |  |  | 3750 | -0.729 | -0.4699 | Yes |
| 65 | BCL11A |  |  | 3756 | -0.735 | -0.4600 | Yes |
| 66 | SOX4 |  |  | 3797 | -0.786 | -0.4580 | Yes |
| 67 | MAPK10 |  |  | 3798 | -0.787 | -0.4461 | Yes |
| 68 | PFN2 |  |  | 3841 | -0.832 | -0.4439 | Yes |
| 69 | CCDC88A |  |  | 3854 | -0.848 | -0.4340 | Yes |
| 70 | SLAIN1 |  |  | 3862 | -0.855 | -0.4227 | Yes |
| 71 | ZEB2 |  |  | 3876 | -0.894 | -0.4124 | Yes |
| 72 | CADPS |  |  | 3877 | -0.894 | -0.3988 | Yes |
| 73 | RBP1 |  |  | 3878 | -0.898 | -0.3851 | Yes |
| 74 | CHD7 |  |  | 3880 | -0.904 | -0.3716 | Yes |
| 75 | MAF |  |  | 3893 | -0.930 | -0.3605 | Yes |
| 76 | SP9 |  |  | 3898 | -0.942 | -0.3472 | Yes |
| 77 | RAB3IP |  |  | 3940 | -1.037 | -0.3416 | Yes |
| 78 | DCLK2 |  |  | 3945 | -1.043 | -0.3268 | Yes |
| 79 | SOX2 |  |  | 3953 | -1.069 | -0.3123 | Yes |
| 80 | SOX6 |  |  | 3956 | -1.081 | -0.2963 | Yes |
| 81 | NEDD4L |  |  | 3960 | -1.090 | -0.2805 | Yes |
| 82 | NCAM1 |  |  | 3966 | -1.106 | -0.2649 | Yes |
| 83 | SOX11 |  |  | 3977 | -1.147 | -0.2500 | Yes |
| 84 | TCF4 |  |  | 3984 | -1.173 | -0.2336 | Yes |
| 85 | CDH2 |  |  | 3998 | -1.233 | -0.2181 | Yes |
| 86 | TUBB3 |  |  | 4009 | -1.334 | -0.2003 | Yes |
| 87 | DPYSL3 |  |  | 4012 | -1.349 | -0.1803 | Yes |
| 88 | ZBTB20 |  |  | 4024 | -1.407 | -0.1617 | Yes |
| 89 | LSAMP |  |  | 4028 | -1.442 | -0.1405 | Yes |
| 90 | DLX1 |  |  | 4041 | -1.558 | -0.1198 | Yes |
| 91 | DLX2 |  |  | 4055 | -1.704 | -0.0971 | Yes |
| 92 | PLXNA4 |  |  | 4060 | -1.752 | -0.0715 | Yes |
| 93 | DLX5 |  |  | 4090 | -2.619 | -0.0389 | Yes |
| 94 | DCX |  |  | 4091 | -2.671 | 0.0017 | Yes |
Table: GSEA details [plain text format]

  

Fig 2: RICHARDS\_NOWAKOWSKI\_SCIENCE\_2017\_NIN1\_UPREG: Random ES distribution      
 Gene set null distribution of ES for **RICHARDS\_NOWAKOWSKI\_SCIENCE\_2017\_NIN1\_UPREG**
